# Picture naming: Stereoelectroencephalography and connectivity insights

**DOI:** 10.1002/epi.70283

**Published:** 2026-05-12

**Authors:** Insafe Mezjan, Fabien Rech, Olivier Aron, Louis Maillard, Sophie Colnat‐Coulbois

**Affiliations:** ^1^ Service de Neurochirurgie, Centre Hospitalier Régional Universitaire (CHRU)‐Nancy Université de Lorraine Nancy France; ^2^ Ingénierie Moléculaire, Cellulaire et Physiopathologie (IMoPA), Centre national de la recherche scientifique (CNRS) Université de Lorraine Nancy France; ^3^ Centre de recherche en automatique de Nancy (CRAN), CNRS Université de Lorraine Nancy France; ^4^ Service de Neurologie, CHRU‐Nancy Université de Lorraine Nancy France

**Keywords:** basal temporal language area, brain mapping, direct electrical stimulation, language, stereoelectroencephalography

## Abstract

**Objective:**

This study investigates the neural substrates of picture naming (PN) using stereoelectroencephalographic (SEEG) stimulations and evaluates the contribution of white matter (WM) fascicles related to the basal temporal language area (BTLA) to the broader functional PN network.

**Methods:**

In patients undergoing SEEG for drug‐resistant epilepsy, stimulation sites were classified as either eloquent (inducing anomia) or noneloquent (NE). Spatial distribution was assessed along the ventral temporal cortex (VTC). Structural connectivity was analyzed using voxelwise disconnectome mapping, connectivity profiles were compared between eloquent and NE sites, and Uniform Manifold Approximation and Projection (UMAP) was applied to characterize the organization of disconnectomes associated with eloquent sites.

**Results:**

Eloquent sites were predominantly located along the VTC, following an anteroposterior gradient, with the highest probability in the posterior fusiform gyrus. These sites showed the strongest overlap with the inferior longitudinal fasciculus (ILF), and eloquent‐site disconnectomes primarily involved the ILF and inferior fronto‐occipital fasciculus. Voxelwise comparisons revealed distinct network profiles between eloquent and NE sites, characterized by higher inferred connectivity in anomia‐derived disconnectomes. UMAP demonstrated an anteroposterior organization of disconnection patterns, reflecting structural heterogeneity across the BTLA.

**Significance:**

PN appears to depend on a ventral temporo‐occipital network organized along an anteroposterior gradient within the VTC, where naming performance may rely more on the integrity of ventral WM pathways than on cortical location alone. The BTLA emerges as a structurally heterogeneous region shaped by the progressive overlap of WM pathways from anterior to posterior temporal regions. These results support integrating SEEG mapping and individualized ventral WM assessment into surgical planning.

**Key points**

- Anomia sites followed an anteroposterior gradient along the ventral temporal cortex, peaking in the mid–posterior fusiform gyrus.
- Anterior anomia sites also exist and should be considered at the individual‐patient level.
- Anomia ROIs showed greater overlap with the left inferior longitudinal fasciculus, supporting a key role for ventral white matter in picture naming.
- Disconnectomes from anomic sites involved ventral temporo‐occipital and frontal networks, whereas noneloquent sites involved perisylvian regions.
- UMAP identified three disconnectome patterns across the anteroposterior axis of the ventral temporal cortex, revealing BTLA heterogeneity along this gradient.

## 1 INTRODUCTION

Temporal lobe epilepsy surgery is effective, with seizure freedom achieved in 72.7% of patients after anterior temporal lobectomy.^1^, ^2^ However, postoperative naming decline is reported in approximately 34% of cases.^3^ Beyond seizure freedom, the goal of epilepsy surgery is now to secure seizure control while also preserving and, ideally, improving cognitive functions. Notably, postoperative improvement in picture naming (PN) has been reported in up to 23.5% of patients.^4^

For temporal lobe epilepsy, recent research has focused on the basal temporal language area (BTLA), which appears to be critical for naming.^5^ The BTLA was first described by Lüders et al. in 1986 as a region in the fusiform gyrus (FG), 1–9 cm from the temporal pole, reliably producing anomia when stimulated with a subdural grid.^6^, ^7^ Later descriptions included an extension to the parahippocampal gyrus (PHG), the inferior temporal gyrus (ITG), and the occipitotemporal sulcus.^8^, ^9^, ^10^ The BTLA has therefore been delineated through focal electrical stimulations that transiently disrupt naming, effectively reproducing the effects of a brief, localized functional lesion.^5^, ^11^, ^12^

Krauss et al. reported naming decline after resection of BTLA sites, whereas patients with spared BTLA sites presented an improvement.^13^ This finding was further supported by a stereoelectroencephalographic (SEEG) study reporting that 57% of patients with BTLA sites resected presented an early PN decline compared with only 9% when BTLA sites were spared.^5^ At 2 years, this difference between groups in decline did not statistically persist, highlighting the possibility of partial long‐term recovery.^5^

In parallel, language studies have moved beyond a localizationist approach, recognizing instead that language processing relies on dynamic interactions across widespread neural networks that encompass both cortical regions and subcortical structures interconnected through white matter (WM) pathways.^14^, ^15^ Within this framework, a cortical or subcortical site that elicits anomia during SEEG stimulation should not be viewed as an isolated locus, but rather as an input gate into a broader functional network supporting PN.^16^ Accordingly, WM tracts involved in naming emerge as fundamental in this framework.

The main associative tracts of interest are the inferior longitudinal fasciculus (ILF), inferior fronto‐occipital fasciculus (IFOF), arcuate fasciculus (AF), uncinate fasciculus (UF),^17^ and pulvinotemporal tract (PTT).^18^

Disruption of the ILF has been shown to induce PN decline after surgery.^19^ Its perioperative stimulation during awake glioma surgery systematically induces pure anomia when the temporal pole is not infiltrated by tumor.^20^ Previous studies have demonstrated its role in the retrieval of both proper names^21^ and common nouns,^22^ highlighting its contribution to visual–lexical integration. Alongside the ILF, the IFOF represents another key component of the ventral language pathway. In patients undergoing resections in the language‐dominant hemisphere, postoperative naming decline at 3 months was significantly associated with greater damage to the IFOF and AF.^23^ Anatomically, the IFOF includes projections within its ventral layer, linking inferior frontal regions to posterior temporal (including the posterior FG) and inferior occipital cortices.^24^ These connections are thought to mediate conceptual semantic control within the visual modality.^24^, ^25^

Together, the ILF and IFOF form the ventral stream, supporting visual‐to‐lexical and semantic integration processes.

The AF, although classically assigned to the dorsal phonological pathway, also appears to contribute to lexical retrieval through its temporal and basal temporal projections, thereby bridging phonological and lexical processing.^26^ The AF has long been described as the major WM pathway linking Broca's and Wernicke's areas. However, Giampiccolo and Duffau demonstrated that arcuate fibers project toward the BTLA and suggested that these terminations may mediate lexical world retrieval.^26^

The involvement of the UF in naming is more difficult to demonstrate, as its anterior course and proximity to other WM bundles often obscure its delineation. Its damage has been associated with naming impairments for unique entities.^22^

PTT have been proposed by Maldonado et al. as key substrates of corticothalamocortical loops supporting PN.^18^ Notably, the thalamus, and particularly the pulvinar, have long been implicated in naming deficits, although robust structural evidence has remained limited.^27^ In their multimodal study combining intraoperative stimulation, tractography, and postmortem dissection, the authors identified and provided convergent evidence for four principal contingents: an optic radiation‐like component projecting to the mid–posterior FG, a lateral component projecting to the middle temporal gyrus (MTG), an auditory radiation‐like component projecting to the superior temporal gyrus (STG), and the Arnold proper tract projecting to the temporal pole.^18^ These pathways were hypothesized to support multilevel lexical processing, with the pulvinar acting as a modulatory hub for lexical processing.^18^

We therefore aimed to use SEEG stimulations to investigate the neural substrates of PN and to evaluate the contribution of WM fascicles related to the BTLA to the broader functional network supporting PN. We further analyzed structural connectivity of eloquent sites using disconnectome mapping,^28^ compared network profiles between eloquent and noneloquent (NE) sites, and characterized the overall organization of ventral temporal connectivity supporting PN.

## 2 MATERIALS AND METHODS

### 2.1 Population

This monocentric, retrospective observational study included 66 patients with drug‐resistant left temporal lobe epilepsy who underwent an SEEG exploration enabling the identification of left anomia sites in the left ventral temporal cortex (VTC). All patients were treated at Centre Hospitalier Régional Universitaire de Nancy, France, a French reference center for rare epilepsies and a tertiary epilepsy surgery center, between January 2007 and December 2023. Patients with a right or bilateral BTLA were excluded. For patients with multiple SEEG procedures, only data from the first implantation, prior to any cortical resection, were analyzed.

### 2.2 SEEG surgical procedure

All SEEG procedures were performed under general anesthesia. Intracerebral electrodes (MICRODEEP, Dixi Medical) were stereotactically implanted using the Leksell Stereotactic System (Elekta). Each electrode had a diameter of .8 mm and consisted of 5–18 contacts (2 mm length, 1.5 mm intercontact spacing). SEEG exploration lasted 1 week; if no seizures were recorded, monitoring was extended into the following week. Number and placement of electrodes was decided during a planification session between the neurosurgeon and the neurologist, depending on the hypothesis of the epileptogenic zone and to map functionally relevant regions. Along the VTC, patients generally had one electrode implanted in the temporal pole, at least two electrodes sampling the anterior and middle VTC, and in some cases, an additional electrode sampling the most posterior portion of the FG. The exact localization of these electrodes varied depending on the vascular constraints.^11^

### 2.3 SEEG stimulations

Anomia sites were defined as the sites where the electrical stimulation of two contiguous contacts (bipolar stimulation, 50 Hz, 5–10 s, .5 to 2 mA) elicited at least one anomia event. Reproducibility was systematically assessed, and sham stimulation were delivered in some patients. Visual object naming was assessed with the Test de dénomination orale d'images 80, which involves the naming of 80 black‐and‐white drawings of objects.^29^ Sites where an afterdischarge was observed or a seizure was triggered were discarded from the analysis. All stimulations were conducted at least 2 h after the last seizure to avoid potential confounding effects of postictal language dysfunction.

### 2.4 Region of interest creation and overlaps

All electrodes tested for PN during SEEG exploration, whether or not they provoked anomia, were identified using Brainstorm^30^ under MATLAB (http://neuroimage.usc.edu/brainstorm). Montreal Neurological Institute (MNI) coordinates were then obtained for each electrode contact. For each stimulation, midpoint coordinates were calculated as the average of the two contacts' coordinates. For each stimulation, we created a spherical region of interest (ROI) with a 1‐cm diameter centered on the midpoint coordinates. Koessler et al. showed that during SEEG stimulation, 75% of the current flow is contained within a cube with a 13‐mm side length and 95% within a 20‐mm‐sided cube.^31^ Thus, our chosen ROI provided a conservative representation of the volume of tissue activated and was selected to maximize specificity.

For each patient, ROIs were binarized (1 = stimulated, 0 = nonstimulated) to prevent artificial hot spots arising from the overlap of adjacent stimulation sites. Using custom Python scripts, we first generated overlap maps of all stimulation ROIs and then created a ratio map (by dividing the number of anomia sites by the total number of stimulations delivered at each voxel) to account for both the spatial distribution of electrodes and the frequency of stimulation within each region. Because electrode implantation was relatively consistent across our cohort, certain regions were more frequently sampled than others, introducing a spatial bias in stimulation density.

We compared the atlas‐derived probability of tract overlap with stimulation ROIs and the occurrence of anomia, using population‐based tractography from the Atlas of Yeh.^32^ This atlas was constructed using the Human Connectome Project (HCP) young adult data from 1065 subjects, available at (https://www.humanconnectome.org/). For each ROI, we computed the mean tract probability across voxels within the sphere for the ILF, IFOF, AF, and UF. We additionally analyzed the overlap of stimulation ROIs with the PTT described by Maldonado et al. using the tract NIfTI reconstructions provided by the authors.^18^ Group differences in mean tract probabilities between NE and anomic ROIs were assessed with the Mann–Whitney *U*‐test.

### 2.5 Disconnectomes

Direct high‐frequency electrical stimulation transiently disrupts brain function when electrical currents are applied to cortical or subcortical regions while patients perform tasks.^16^, ^26^ We hypothesized that such stimulations emulated a transient functional disconnection, disrupting not only the local cortical area but also the WM pathways connected to it. To characterize these network‐level effects, disconnectome maps were generated for each stimulation site (ROI) using DeepDisco (https://github.com/annamatsulevits/DeepDisco)^33^ for associative fibers. Comparison between disconnectomes derived from anomic stimulation sites and NE stimulation sites were performed using a voxelwise permutation analysis of general linear models from FMRIB Software Library (https://fsl.fmrib.ox.ac.uk/fsl/docs/index.html).^34^ A two‐sample unpaired *t*‐test design was used to identify WM regions showing differential disconnection patterns between anomic stimulation sites and NE stimulation sites. Two one‐sided contrasts were computed to test for greater disconnectivity in anomic versus NE sites and the reverse comparison. Significance testing was performed using Threshold‐Free Cluster Enhancement with 5000 permutations.

Visualizations were generated in MRIcroGL (https://www.nitrc.org/projects/mricrogl).

### 2.6 Uniform Manifold Approximation and Projection

To reduce the dimensionality of the eloquent disconnectome maps, we used the Uniform Manifold Approximation and Projection (UMAP) algorithm implemented in Python.^35^ UMAP estimates similarities between disconnectome profiles and embeds them into a third‐dimensional space based on the Euclidean distance between each disconnectome. To aid interpretability, each point was color‐coded according to the anteroposterior (y‐axis) MNI coordinate of the corresponding stimulation‐derived ROI that generated the disconnectome, thereby linking the spatial anatomical location of the original stimulation site to the UMAP representation of its corresponding disconnectome.

All statistical analyses were performed using Jamovi version 2.5.6 (the Jamovi project). For all statistical analyses, the level of significance was set at *p* < .05.

This study was performed in accordance with the ethical standards laid down in the 1964 Declaration of Helsinki and its later amendments. It was approved by the institutional review board of the Collège de Neurochirurgie (no. IRB00011687).

## 3 RESULTS

### 3.1 Population studied

Sixty‐six patients were enrolled in the study, comprising 41 women (62.1%). Mean age at first seizure was 17.8 ± 11 years (range = .16–44). Mean age at SEEG was 33.3 ± 9.22 years (range = 14–53). Median duration of epilepsy before SEEG was 13.5 years (interquartile range [IQR] = 7.25–19.8). Most patients were right‐handed (87.9%); four (6.1%) were left‐handed, and four (6.1%) were ambidextrous. Mean level of education was 13 ± 3.16 years (range = 5–21). A total of 63.6% of patients were employed or enrolled in studies. The number of antiepileptic drugs at the time of SEEG ranged from 1 to 4, with a median of 2 (IQR = 2–3). Magnetic resonance imaging (MRI) abnormalities were present in 59.1% of patients.

### 3.2 Stimulations

A total of 1937 stimulation sites were analyzed, including 376 eloquent (ROIs associated with electrically induced anomia) and 1561 NE ROIs. Across all 66 patients, the mean number of stimulation sites tested for naming was 29.3 ± 13.5 (range = 4–61). The mean number of eloquent stimulation sites per patient was 5.7 ± 3.49 (range = 1–15), and mean number of NE stimulation sites was 23.7 ± 12.5 (range = 11–56). Stimulation intensities were comparable between eloquent and NE sites (*p* = .197) with a median intensity of 1.2 mA in both groups (.5–3.0 mA for NE sites and .6–2.2 mA for eloquent sites).

**FIGURE 1:**
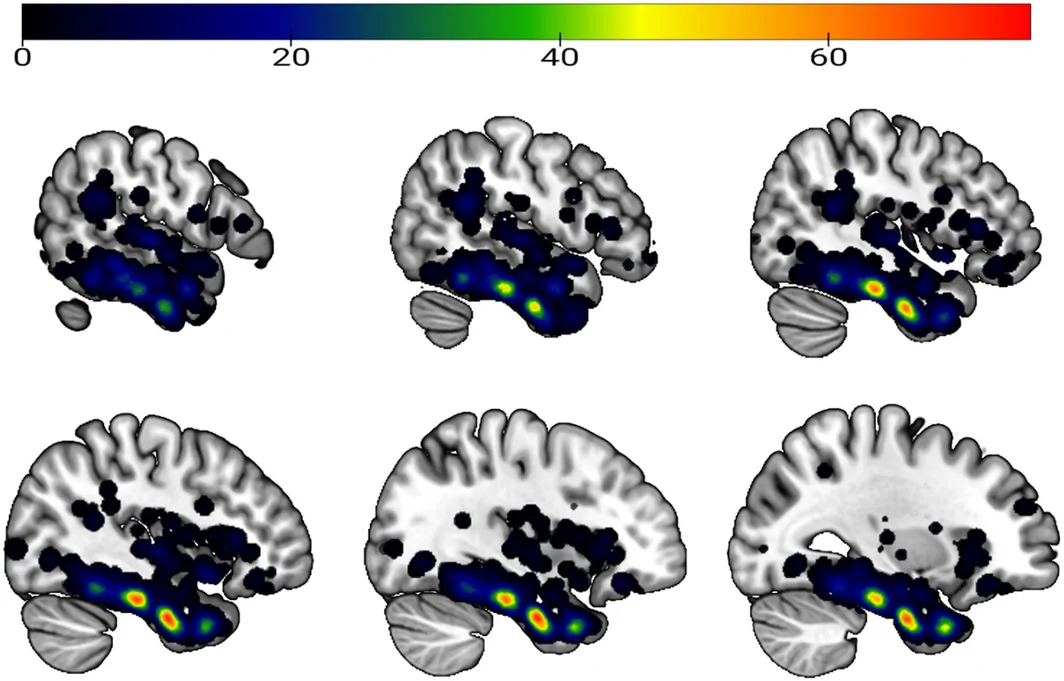
Overlap map of all stimulation sites tested for anomia during stereoelectroencephalography (*n* = 1937). The color bar indicates the number of overlapping regions of interest across patients.

Overlay of all stimulation sites tested for anomia (Figures 1 and S1) revealed a broad distribution across the inferior frontal cortex, the entire temporal lobe, and the angular and supramarginal gyri.

Eloquent sites spanned the entire extent of the VTC, from the most anterior site located at x = −24, y = 17, z = −39 to the most posterior site at x = −42, y = −52, z = −24, corresponding to an anteroposterior distance of 69 mm (Figure S2).

**FIGURE 2:**
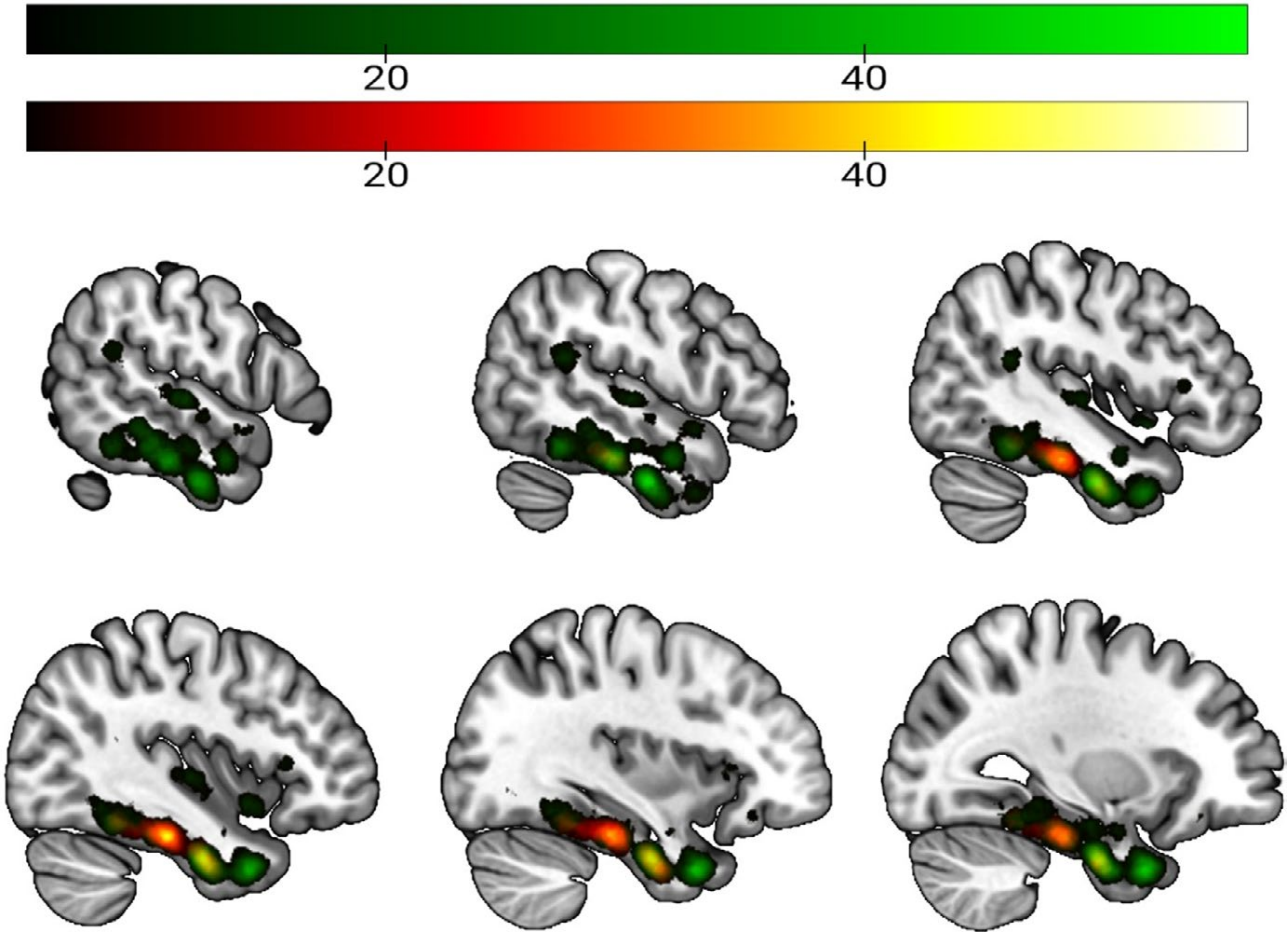
Overlap maps of eloquent (*n* = 376; red–yellow gradient) and noneloquent stereoelectroencephalographic stimulation sites (*n* = 1561; green gradient). Additive blending demonstrates an anteroposterior gradient, with a higher density of eloquent sites in posterior regions. The color bar indicates the number of overlapping regions of interest across patients. Display threshold: ≥5.

Overlapping the eloquent and NE stimulation sites revealed a clear anteroposterior gradient along the VTC (Figure 2), with a higher density of eloquent sites posteriorly. Anterior eloquent sites were present but exhibited lower and less consistent probabilities. Anomia probability peaked in the posterior FG and progressively declined anteriorly.

### 3.3 Ratio map of anomia

**FIGURE 3:**
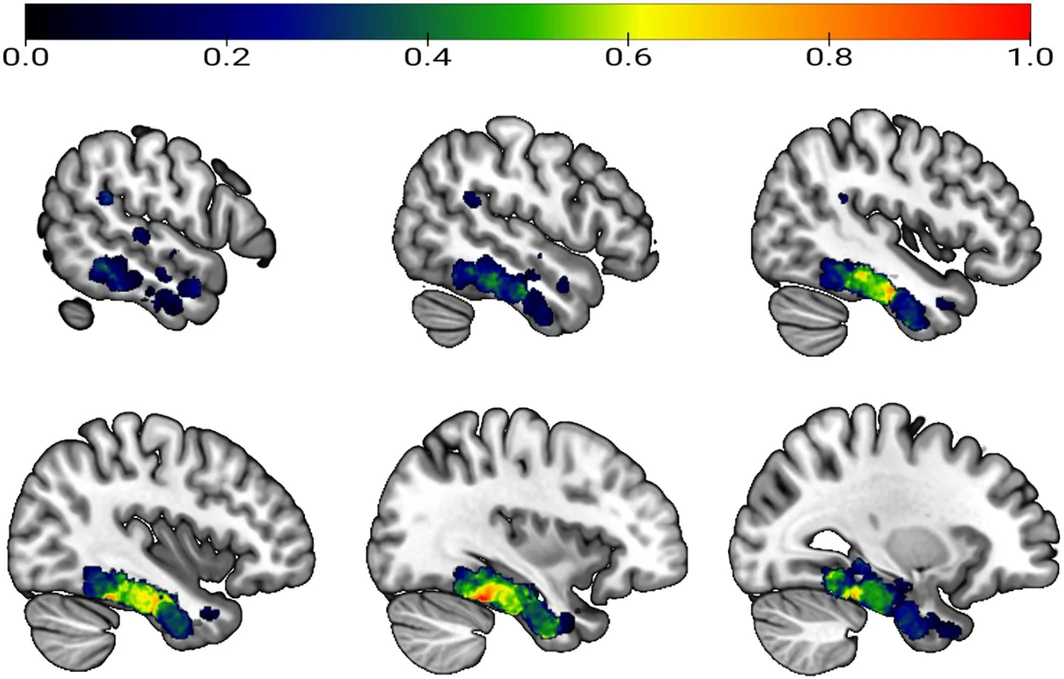
Gradient map of anomia probability. The color bar indicates the probability (0–1) of eliciting anomia upon stimulation. Threshold: ≥5 overlapping stimulations.

The ratio map of anomia provided a normalized estimate of the probability of eliciting anomia independent of sampling density (Figures 3 and S3). This map revealed an anteroposterior gradient with peak anomia probabilities in two regions: a first hotspot in the FG and ITG (x = −42.5, y = −18.5, z = −24.5) and a second at the posterior aspect of the FG (x = −33, y = −39, z = −24).

### 3.4 Disconnectome maps

**FIGURE 4:**
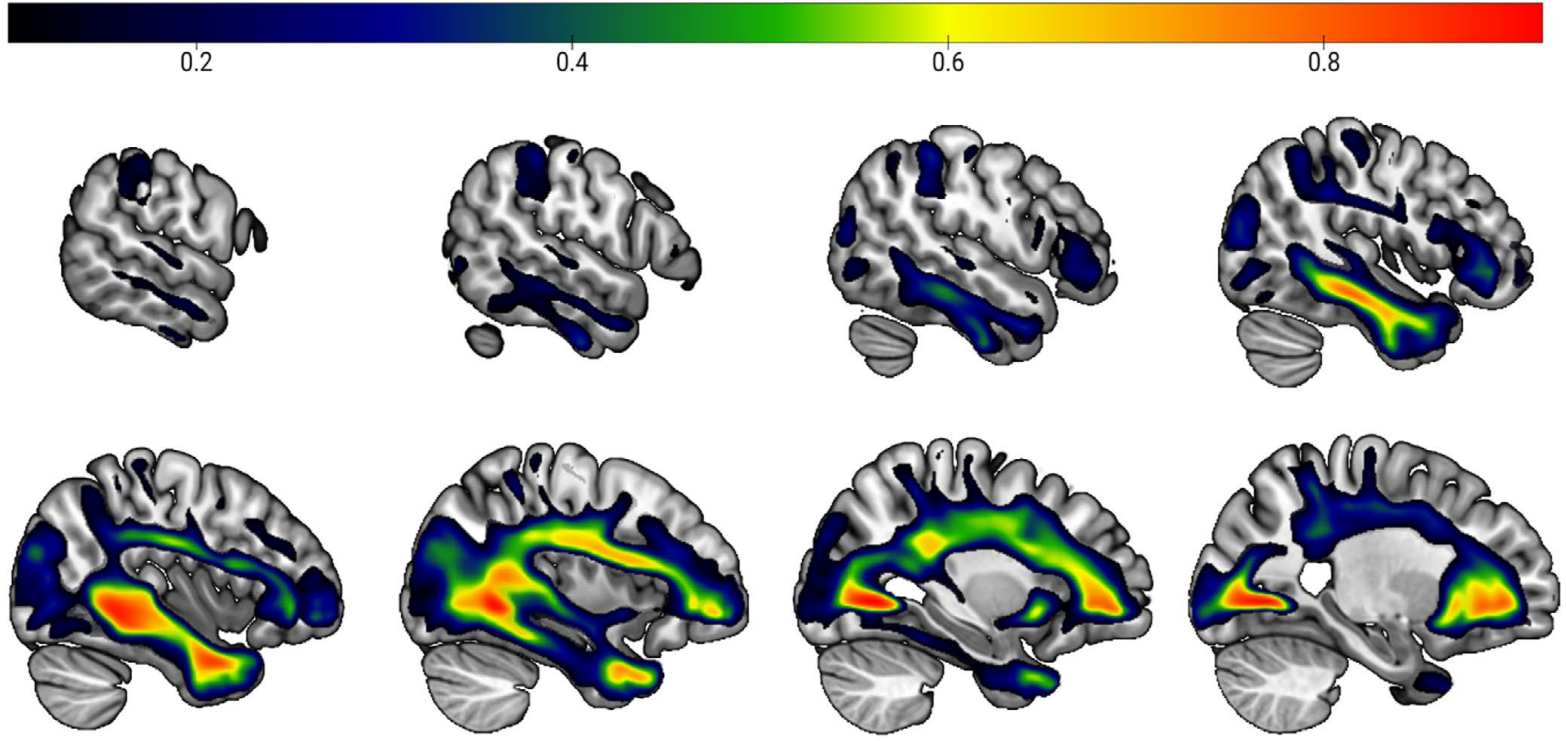
Map showing the mean probability of disconnection (0–1 on the color bar) across all 376 eloquent stimulation‐derived regions of interest.

Disconnectome maps derived from eloquent ROIs showed involvement of associative WM tracts, mainly the ILF and the IFOF, with additional but less prominent contributions from the AF, UF, and frontal aslant tract (FA; Figures 4 and S4). In contrast, disconnectomes generated from NE ROIs showed a more spatially restricted extent of involvement of the ILF, IFOF, AF, UF, and FA. Under the caveat that thalamic terminations were not visible in our disconnectome maps, the optic radiation‐like component of the PTT, the lateral PTT component, and the Arnold proper component may represent additional contributions to the disconnectome maps derived from eloquent ROIs, and to a more discrete extent in the NE ROI‐derived disconnectomes.

### 3.5 WM fascicles and anomia ROIs overlap

When looking for correlations between the overlap of anomia ROIs and the probability of WM fascicles (HCP), overlap with the left ILF was significantly higher for anomia ROIs than NE ones (median = .11 vs. .022, Mann–Whitney *p* < 10^−24^, area under the curve [AUC] = .672, *r* = .345). We found no evidence of difference for the AF or the IFOF (*p* = .805 and *p* = .758). UF showed an inverse direction effect, with anomia ROIs having a lower overlap with the tract than NE ROIs (*p* < 10^−9^, AUC = .413, *r* = −.172).

When examining overlap between anomia ROIs and PTT components, the optic radiation‐like segment showed significantly greater overlap for anomia than NE ROIs (median = .00163 vs. 7.68 × 10^−5^, Mann–Whitney *p* < 10^−45^, AUC = .730, *r* = .460). In contrast, the auditory radiation‐like segment and the Arnold proper tract showed lower overlap in anomia ROIs (respectively, median = .0 vs. .0, *p* < 10^−7^, AUC = .447, *r* = −.105 and median = 7.34 × 10^−5^ vs. 6.98 × 10^−4^, *p* < 10^−6^, AUC = .418, *r* = −.164). No evidence of a difference was observed for the lateral segment (*p* = .223, AUC = .516).

### 3.6 Disconnectome contrast analysis

**FIGURE 5:**
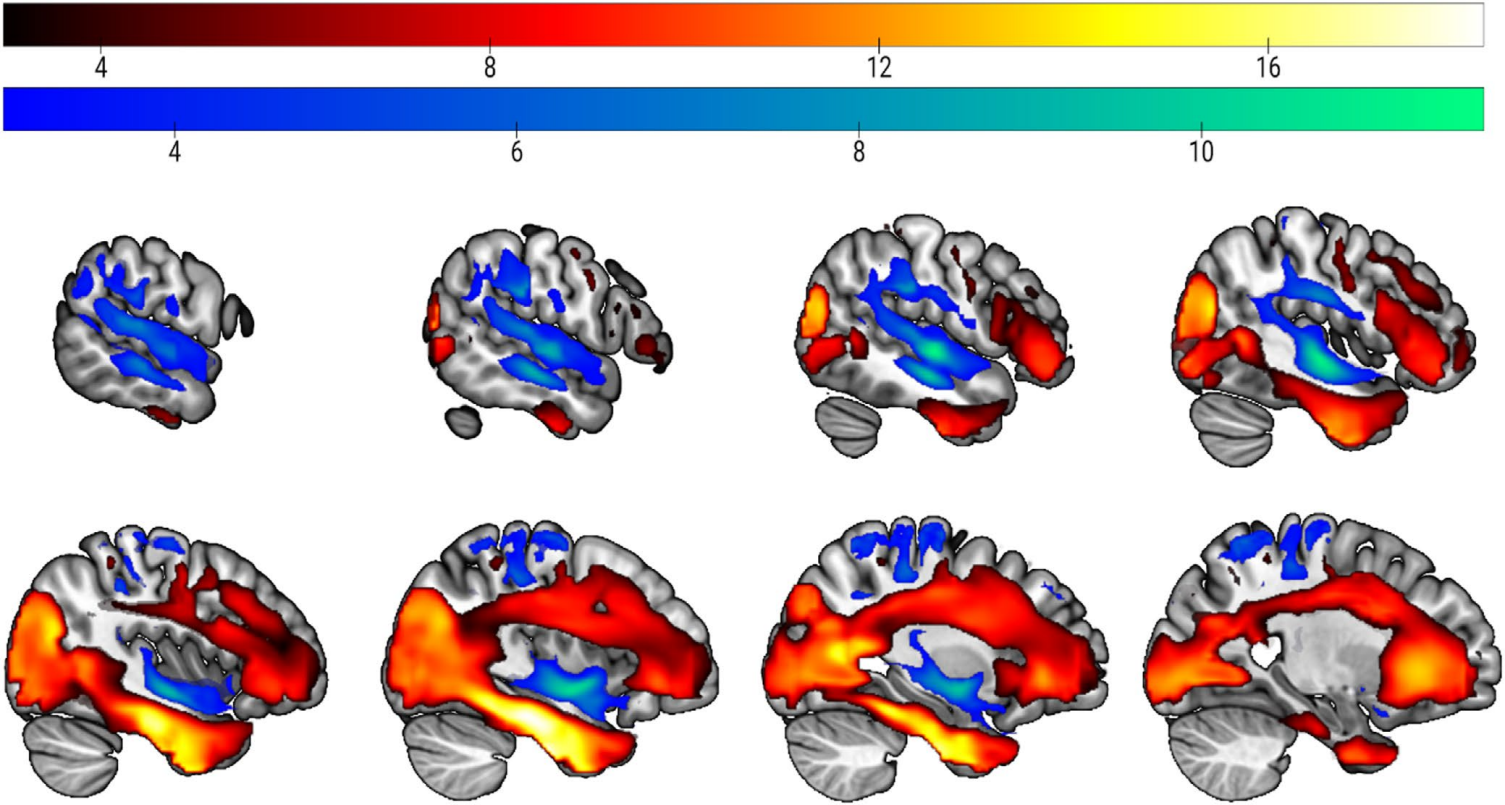
Voxelwise comparison of disconnectome maps between anomic and noneloquent stimulation sites. Regions with greater disconnectivity in anomic (eloquent) region of interest (ROI)‐derived disconnectomes are displayed in red–yellow, whereas regions with greater disconnectivity in noneloquent ROI‐derived disconnectomes appear in blue–green. Color bars indicate *t*‐values from the voxelwise comparison. Only voxels surviving Threshold‐Free Cluster Enhancement‐corrected significance (*p* < .05) are displayed.

The resulting 1937 ROI‐derived disconnectome maps, representing voxelwise probabilities of WM disconnection, were compared between anomic and NE stimulation sites. The resulting map (Figure 5) revealed greater involvement of the VTC, including the ITG, FG, and PHG, along with the occipital lobe, inferior frontal cortex, and cingulum in disconnectomes derived from anomic sites. In contrast, regions showing stronger connectivity in NE ROI‐derived disconnectomes were predominantly located in the STG, MTG, angular and supramarginal gyri, insula, and pre‐ and postcentral regions.

### 3.7 Uniform Manifold Approximation and Projection

**FIGURE 6:**
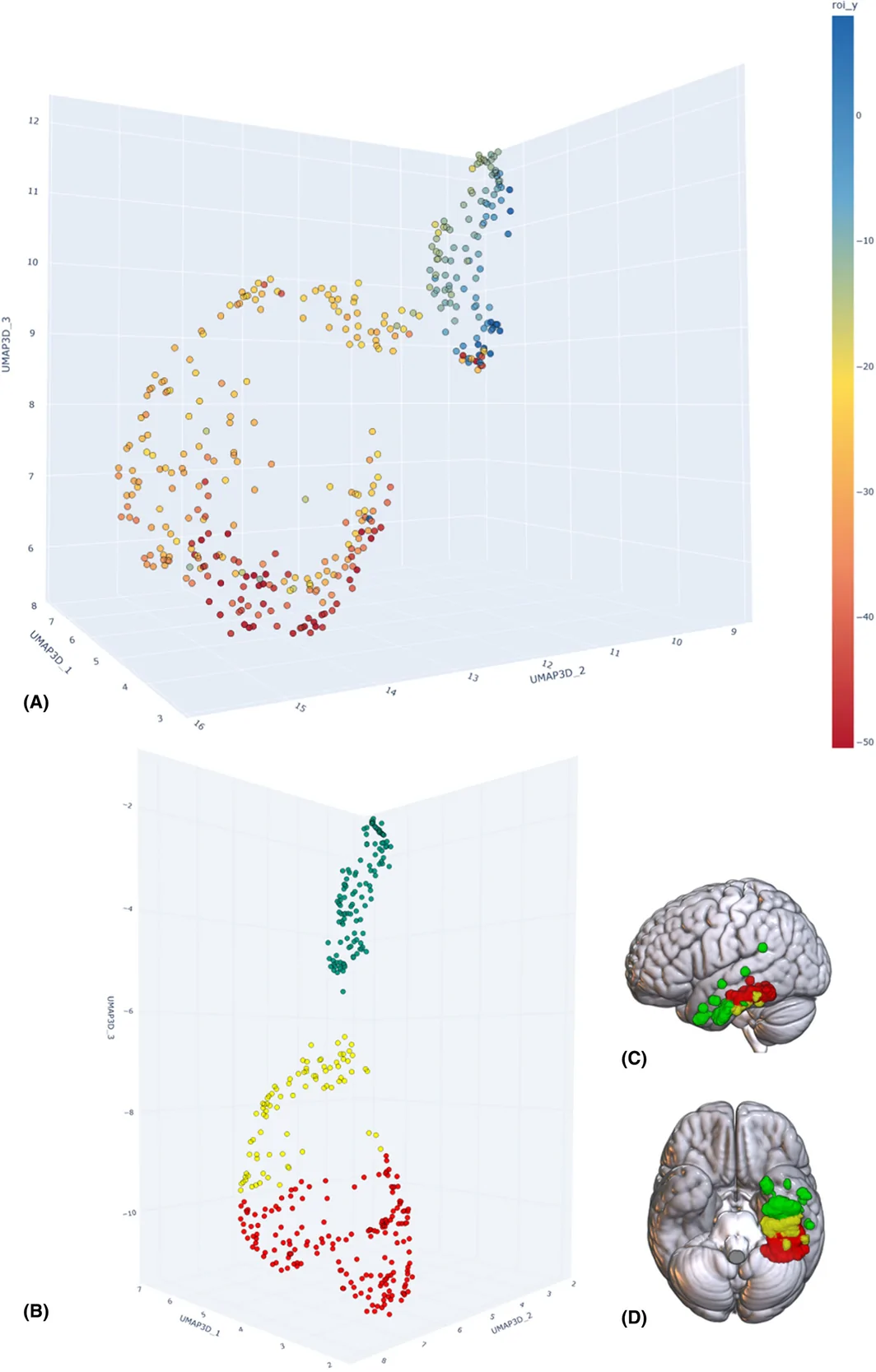
(A) Uniform Manifold Approximation and Projection (UMAP) embedding of disconnectome maps derived from anomic stimulation sites, showing a robust intrinsic segregation of disconnection profiles. Each point represents a stimulation–region of interest (ROI) disconnectome and is color‐coded according to the anteroposterior (Montreal Neurological Institute y‐axis) coordinate of the originating ROI, revealing a continuous spatial gradient along the ventral temporal cortex. (B) Data‐driven clustering of the UMAP embedding identifies three clusters of disconnectome profiles (red, green, yellow). (C) Left sagittal cortical surface rendering of the eloquent stimulation sites, color‐coded according to their respective UMAP disconnectome‐derived clusters, illustrating their anteroposterior progression. (D) Inferior cortical surface rendering of the same eloquent stimulation sites, color‐coded according to their respective clusters, highlighting the ventral spatial distribution of the three clusters.

The UMAP embedding of anomic stimulation‐derived disconnectomes revealed an intrinsic organization of disconnectome profiles, with distinct clusters emerging spontaneously without any imposed spatial constraint. When each point was color‐coded according to the anteroposterior (y‐axis) MNI coordinate of the corresponding ROI, a clear gradient became apparent (Figure 6). The first cluster, comprising 117 ROIs (mean y = −9.78), was located along the temporal pole and the most anterior portions of the FG and PHG. The second cluster (66 ROIs, mean y = −25.20) involved the anterior FG and PHG, whereas the third and largest cluster (193 ROIs, mean y = −33.71) extended along the mid–posterior FG and PHG, reflecting a continuous anteroposterior organization of basal temporal disconnection patterns underlying anomia.

## 4 DISCUSSION

In this study, we investigated the cortical and subcortical substrates of PN using SEEG stimulations as a framework for transient functional perturbation. Stimulations eliciting anomia were predominantly located along the VTC, following an anteroposterior gradient, with the highest probability of anomia in the posterior FG and progressively lower probabilities anteriorly. Eloquent stimulations showed a prominent overlap with the left ILF, underscoring its role in PN. Considering that SEEG stimulation likely transiently disturbs the adjacent WM tracts, voxelwise disconnectome comparisons suggested distinct network profiles between eloquent and NE sites, characterized by higher inferred connectivity in anomia‐derived disconnectomes. Finally, UMAP‐based dimensionality reduction demonstrated a distinct anteroposterior organization of disconnection patterns along the basal temporal axis, highlighting the structural heterogeneity of this region.

In our study, the BTLA was identified in the FG, ITG, and PHG, spanning 69 mm from the temporal tip, in line with the literature.^5^, ^7^, ^8^, ^13^ When it comes to the anomia ratio map, the anomia hotspots converged with epilepsy studies that used voxel‐based lesion–symptom mapping (VLSM) to identify critical regions for naming, reinforcing the critical role of the mid–posterior FG in PN.^36^, ^37^, ^38^ More recently, Mhanna et al. reported that PN decline was most strongly associated with resection of the BTLA and the left anterior MTG.^39^ Although the MTG was not implicated in our results, both studies converge on the critical involvement of the VTC in naming functions.

Importantly, our results reveal the existence of anterior eloquent sites, which, although less frequent and more variable at the group level, can still be crucial at the single‐patient level. This aligns with previous evidence that resecting anterior BTLA regions increases the risk of postoperative PN decline.^5^ Therefore, recognition of these anterior eloquent regions is essential for defining the posterior limit of resection. When the epileptogenic zone overlaps these regions, either informed patient consent or reconsideration of surgical indication should be carefully discussed.

We have previously shown that postoperative cavities following left temporal lobe epilepsy surgery at our center remain anterior to the cortical regions identified as critical for naming in VLSM analyses, and that a strictly cortical localizationist framework does not account for the language outcomes observed.^4^, ^36^, ^37^, ^38^ These findings are consistent with those of Davies et al., who reported naming decline even after tailored temporal resections guided by extraoperative subdural language mapping.^40^ The presence of postoperative language deficits despite sparing critical cortical regions suggests a disconnection mechanism involving underlying WM pathways and consequently, the integrity of language networks.^17^ The involvement of WM in the function of the BTLA has long been discussed, with Lüders et al. first noting that the WM underlying the BTLA and Wernicke's area are in direct contact.^7^ Corticocortical evoked potential (CCEP) studies have demonstrated robust connectivity between the inferior frontal lobe and the BTLA,^41^ and have showed that the BTLA operates within the ventral stream, predominantly supported by the ILF.^42^

In this study, disconnectome analysis revealed a predominant involvement of the ILF and IFOF, with a more discrete possible contribution from the AF and UF. When comparing voxelwise probabilities of WM disconnection between anomic and NE stimulation sites, regions showing stronger connectivity for eloquent ROIs were mainly located along the VTC and occipital lobe, extending to the frontal lobe. Frontal terminations were most prominent mesially, with additional projections extending laterally into the anterior language area. These patterns are consistent with the trajectories of the ILF, IFOF, and UF, whereas the AF likely accounts for the lateral frontal projections. Interestingly, these disconnection patterns closely mirror the functional activations reported by Forseth et al.,^43^ who observed enhanced blood oxygen level‐dependent activity during PN along the occipital–ventral temporal–frontal continuum, terminating in the middle FG and inferior frontal cortex. This correspondence suggests that the regions structurally connected through the ILF, IFOF, and UF constitute the anatomical substrate of the ventral naming network identified functionally in electrophysiological and functional MRI studies.^43^ Consistent with our findings, Mhanna et al. recently reported that the peak site of lesion connectivity associated with naming decline was located within the temporal stem, at the confluence of the ILF and IFOF.^39^

Conversely, regions showing greater connectivity for NE than eloquent sites were mainly distributed across perisylvian cortices, including the STG, MTG, angular gyrus, and supramarginal gyrus, with only limited mesial involvement, mostly confined to the pre‐ and postcentral gyri. This distribution could correspond to connections of NE sites via the dorsal AF pathway with cortical terminations in the middle STG and MTG, mediating acoustic phonological encoding.^26^ This is in line with early CCEP studies reporting no clear response in the basal temporal area when stimulating the posterior language area, namely the STG and MTG, suggesting weaker functional connectivity compared with anterior language areas.^41^ This also aligns with recent CCEP findings indicating that NE sites within the anterior FG preferentially connect to extra‐VTC regions, such as the STG and MTG.^44^

Interestingly, this distribution mirrors the functional contrasts reported by Forseth et al.,^43^ in which regions more active for scrambled than real images during PN were mainly along the dorsal perisylvian cortex. This suggests that the posterior perisylvian network participates predominantly in phonological processing or articulation rather than lexical–semantic retrieval.^43^ Our findings thus provide the structural counterpart of these functional results, showing that regions structurally connected through the ventral temporal pathways likely support PN, whereas the posterior perisylvian cortex, more connected through dorsal tracts, is less involved in PN.

When interpreting our findings in light of the PTT described by Maldonado et al.,^18^ the main limitation is that our disconnectome maps do not capture thalamic terminations, which may lead to an underestimation of PTT involvement. Despite this limitation, distinct patterns emerged across the tract components.

The optic radiation‐like component showed the strongest overlap with eloquent ROIs. The tract showed a spatially concordant trajectory with the disconnectome map of eloquent ROIs. In the voxelwise disconnectome contrast analysis, the occipital terminations of this tract were significantly more associated with anomic than NE stimulation sites, supporting a preferential association with the anomic network.

The Arnold proper tract showed lower overlap with anomic ROIs than with NE ROIs and projected toward the temporal pole, yielding a trajectory reminiscent of the UF. Nevertheless, the tract showed a spatially concordant trajectory with the disconnectome map of anomic ROIs. Disconnectome contrast analysis further highlighted a strong implication of the temporal pole in anomia, where both the UF and the Arnold proper tract exhibit cortical terminations.

The lateral component showed no significant overlap with anomic ROIs in our analysis, although it is described as coursing near the BTLA.^18^ However, the tract exhibited a trajectory spatially concordant with the disconnectome map of anomic ROIs. Moreover, its terminations were located laterally, in proximity to the anomic stimulation sites in our cohort, and disconnectome contrast analysis indicated that these cortical terminations fall within regions more strongly connected to anomic sites than to NE sites.

In contrast, the auditory radiation‐like component did not emerge in the ROI overlap analysis and did not show spatial concordance with the disconnectome map of eloquent ROIs. Its known cortical terminations in the STG^18^ lie within regions more strongly connected to NE sites in the disconnectome contrast analysis. This pattern is consistent with the proposed role of this pathway in lexicophonological processing.

Finally, our UMAPs show distinct disconnection patterns across the anteroposterior axis of the VTC. The anterior cluster, encompassing the temporal pole and the most rostral portions of the FG and PHG, may reflect a disconnection pattern predominantly involving the UF, the Arnold proper tract of the PTT, and the most anterior fibers of the ILF. The second cluster, involving the anterior FG and PHG, may represent an intermediate zone along this ventral pathway, where the ILF is predominantly involved, with additional participation from the lateral component of the PTT. The third and largest cluster, encompassing the mid–posterior FG and PHG, likely corresponds to the core of the BTLA, where the highest probabilities of anomia were observed in our ratio maps. This region, extending across the VTC, likely reflects the joint involvement of the ILF and AF, whose intersection forms the temporo‐occipital bottleneck critical for visual–semantic–lexical integration. The optic radiation‐like component of the PTT may also contribute in this region. Notably, the third cluster encompasses both anterior and posterior ROIs that exhibit a comparable dysconnectivity profile, suggesting that anterior regions may be structurally as relevant as posterior sites. Our hypothesis is that the gradient of the UMAP is driven by the progressive addition and overlap of WM pathways from anterior to posterior temporal regions.

This disconnectome gradient could also help explain the variable postoperative PN outcomes and the inconsistent findings reported after resection of BTLA sites.^5^, ^7^, ^11^, ^13^, ^36^, ^37^, ^38^ The most anterior sites, located near the temporal pole, are likely mediated by the UF, whose indispensability for naming remains debated,^45^ and the Arnold proper tract. Resection of these anterior portions may therefore lead to transient or minimal naming decline, reflecting the potential for compensatory reorganization within the naming network. In contrast, posterior BTLA regions likely lie at the intersection of the ILF and AF within the temporo‐occipital bottleneck, with potential additional involvement of the lateral and the optic radiation‐like components of the PTT. Resection at this level would simultaneously disrupt multiple association systems, markedly reducing the probability of functional recovery. Consequently, the heterogeneity of naming outcomes following BTLA resection likely depends on which combination of WM tracts is affected. However, at the individual‐patient level, this anterior–posterior distinction is not absolute. The precise location of the BTLA varies across patients, as do the terminations, projections, and dominance of each tract within the VTC. Thus, it cannot be generalized that anterior resections are safe whereas posterior ones are not. Instead, the risk for postoperative anomia likely depends on the patient‐specific configuration of ventral WM pathways. Future studies combining individualized tractography with functional mapping will be essential to determine, for each patient, which fibers are critical and which can be safely resected.

Finally, although our findings emphasize the role of the BTLA and its ILF connectivity in PN, they should be interpreted within the broader contemporary framework of the ventral anterior temporal lobe semantic hub. Increasing evidence indicates that this region is not a language‐specific module dedicated solely to naming, but rather forms part of the bilateral system described by Lambon Ralph et al., which functions as a transmodal conceptual hub supporting semantic processing across input modalities.^46^ Within this model, the BTLA is best understood as a connectivity‐defined subregion of the hub whose disruption preferentially affects PN in the dominant hemisphere, such that anomia represents only one clinical manifestation of conceptual system dysfunction. The graded functional architecture of this semantic hub is thought to be shaped by the differential terminations of long‐range WM pathways and provides a unifying account consistent with our UMAP‐derived clusters demonstrating distinct connectivity profiles across the VTC.^46^

From a complementary disconnection perspective, disruption of the ILF has been associated with a broader spectrum of modality‐specific agnosias, including visual hemiagnosia,^47^ alexia, and prosopagnosia,^48^ suggesting that anomia may represent the language‐dominant expression of ventral temporal network dysfunction. Taken together, our findings support a model in which the BTLA is not an isolated site but rather a functionally embedded node within a distributed, connectivity‐defined semantic hub.

This study has several limitations. First, the estimation of the volume of tissue activated by the SEEG stimulation represents an approximation that may not fully capture the true spatial extent of current spread and its effects on surrounding neural elements. Second, variability in stimulation parameters across sites was not modeled, although such differences can influence both the volume and distribution of activated tissue and the engagement of adjacent cortical and subcortical structures. Future work integrating biophysical modeling will be essential to better characterize the local and network‐level effects of SEEG stimulation. Third, individual patient tractography was not incorporated, limiting the ability to account for interindividual variability in WM architecture and potential epilepsy‐related reorganization. These factors may affect the spatial precision of disconnectome‐based analyses. Finally, language assessment was restricted to PN and therefore does not capture the broader domain of language processing (e.g., proper nouns, auditory naming, semantic category distinctions), limiting the generalizability of the findings to other linguistic functions.

## 5 CONCLUSIONS

Our findings indicate that PN appears to depend on a ventral temporo‐occipital network organized along an anteroposterior gradient within the VTC, in which naming performance may rely more on the integrity of ventral WM pathways rather than cortical location alone. The BTLA emerges as a structurally heterogeneous region, whose functional relevance reflects progressive addition and overlap of WM pathways from anterior to posterior temporal regions. This framework helps explain the variability of naming outcomes after temporal resections and supports incorporating SEEG mapping and individualized ventral WM assessment into surgical planning.

## AUTHOR CONTRIBUTIONS

**Insafe Mezjan:** Writing—original draft; writing—review and editing; software; methodology; investigation; formal analysis; data curation; conceptualization. **Fabien Rech:** Methodology; validation; supervision; writing—review and editing. **Olivier Aron:** Data curation; validation; supervision; writing—review and editing. **Louis Maillard:** Writing—review and editing; validation; supervision; project administration; conceptualization. **Sophie Colnat‐Coulbois:** Writing—review and editing; validation; supervision; project administration; conceptualization.

## CONFLICT OF INTEREST STATEMENT

The authors declare that they have no competing financial interests or personal relationships that could be perceived to have influenced the work reported in this paper. We confirm that we have read the Journal's position on issues involved in ethical publication and affirm that this report is consistent with those guidelines.

## Supporting information


**Figure S1.** Overlap map of all stimulation sites tested for anomia during stereoelectroencephalography (*n* = 1937) shown in axial and coronal views. The color bar indicates the number of overlapping regions of interest across patients.

**Figure S2.** Overlap map of all eloquent stimulation sites inducing anomia (*n* = 376) shown in axial views. The color bar indicates the number of overlapping regions of interest across patients.

**Figure S3.** Gradient map of anomia probability, shown in axial and coronal views. The color bar indicates the probability (0–1) of eliciting anomia upon stimulation. Threshold: ≥5 overlapping stimulations.

**Figure S4.** The mean probability of disconnection (0–1 on the color bar) across all 376 eloquent stimulation‐derived regions of interest, shown in axial and coronal views.

## Data Availability

The data that support the findings of this study are available from the corresponding author upon reasonable request.
